# Evaluation of bladder function in children with familial mediterranean fever and outcomes: A retrospective study

**DOI:** 10.1186/s12894-025-01777-9

**Published:** 2025-04-12

**Authors:** Sevim YENER, Şeyma TÜRKMEN, Mehmet Oğuzhan AĞAÇLI, Zekeriya İLÇE, Betül SÖZERİ

**Affiliations:** 1 Department of Pediatric Urology, Umraniye Training and Research Hospital, Health Sciences University, Adem Yavuz Street, 34764 Umraniye, Istanbul, Türkiye; 2 Department of Pediatric Rheumatology, Umraniye Training and Research Hospital, Health Sciences University, Istanbul, Türkiye; 3 Department of Radiology, Umraniye Training and Research Hospital, Health Sciences University, Istanbul, Türkiye; 4 Department of Pediatric Surgery, Umraniye Training and Research Hospital, Health Sciences University, Istanbul, Türkiye

**Keywords:** Bladder, Familial mediterranean fever, Ultasonography, Uroflowmetry

## Abstract

**Background:**

The aim of this study was to investigate bladder functions in patients diagnosed with familial Mediterranean fever but without urological complaints using non-invasive methods.

**Materials and methods:**

Patients aged between 6 and 17 years diagnosed with FMF were included in the study. Complete urinalysis, ultrasonography (renal parenchymal thickness, hydronephrosis, bladder wall thickness, postmicturition residual urine) were recorded for the patients. Uroflowmetry was performed for the patients.

**Results:**

A total of 51 patients were included in the study, 28 of whom were female (54.9%) and 23 were male (45.1%). The mean age of the patients was 11.8 (± 3.8) years. Hydronephrosis was detected in 3 patients (5.9%) in the ultrasonography. The mean bladder wall thickness measured was 1.14 (± 1.6) mm. Significant postmicturition residual urine (> 20 ml) was recorded in 15 patients. There was a statistically significant positive relationship between uroflowmetry bladder capacity (UFM-BV) values (*p* < 0.001). Normal Q max values were measured in 9 patients (17.6%).

**Conclusion:**

Considering the inflammatory effect of FMF, it is important to evaluate bladder function in asymptomatic patients with parameters measured by uroflowmetry to predict its long-term effects. Additionally, ultrasonographic measurement may be misleading in the evaluation of bladder capacity.

## Introduction

Familial Mediterranean Fever (FMF) is an autosomal recessive autoinflammatory disease characterized by recurrent short-lasting febrile and serositis attacks, often associated with systemic amyloidosis [1]. It has higher prevalences among Jews, Turks, Armenians, and Arabs, ranging from 1/1,000 to 1/250 [2–3]. In a study conducted in our clinic regarding patients with daytime urinary incontinence, we found that FMF was the most common non-urological disease encountered after reasons such as constipation, autism, and hyperactivity [4]. Literature reviews generally include studies on the association of AAA with renal amyloidosis and secondary amyloidosis involving the urinary system. It is well known that the most serious complication of FMF is the development of secondary amyloidosis. Amyloidosis, not a specific entity, develops as a result of similar protein accumulation in organs and tissues, either systemically or localized, due to various diseases [5, 6]. Bladder involvement in amyloidosis is rare; the involvement is mostly limited to the bladder, termed as primary bladder amyloidosis [6]. Secondary bladder amyloidosis has been reported in patients with rheumatoid arthritis, Crohn’s disease, ankylosing spondylitis, multiple myeloma, and familial Mediterranean fever [7]. Besides genetic factors, environmental factors also play a role in the development of this complication. The presence of amyloidosis is the most important determinant of the prognosis of FMF. The frequency of amyloidosis development varies among populations and races, being reported as 12.9% in a study conducted in our country [8]. However, with early diagnosis and early initiation of colchicine therapy, this rate decreases to around 1% [9].

In this study, we focused on patients with Familial Mediterranean Fever (FMF) who were under follow-up but did not present with systemic or renal amyloidosis and had no urological symptoms. A review of the literature revealed no studies specifically investigating bladder function in this patient group. Based on this information, our study aimed to evaluate the bladder function of FMF patients through ultrasonographic assessments (including renal ultrasonography) and uroflowmetric parameters, thereby providing insights into their bladder function.

## Patients and methods

Patients who were under routine follow-up for Familial Mediterranean Fever (FMF) at the pediatric rheumatology clinic between 2022 and 2023, in remission, and without any urinary system complaints were included in the study. The patients were evaluated during their routine check-ups. Patients with additional diseases, neurogenic bladder, spinal cord anomaly, bladder dysfunction or who had undergone surgical procedures affecting the urinary system were excluded from the study. Complete urine analysis, urinary system ultrasonography (including parenchymal thickness of the kidneys, presence/absence of hydronephrosis, bladder wall thickness, postmicturition residual urine measurement) were recorded for all patients. All ultrasonographic examinations were performed by a single radiologist. Free uroflowmetry was performed for all patients. Voided volume, Q max value, and voiding velocity were evaluated. This retrospective study was approved by the Ethics Committee of our hospital (no. B.10.1.TKH.4.34.H.GP.0.01/123). The study was conducted in accordance with the principles of the Helsinki Declaration. Since this study was retrospective in nature, the need for informed consent was waived by the Ethical Committee of our hospital.

## Results

A total of 51 patients aged between 6 and 17 years were included in the study. Among them, 28 (54.9%) were female, and 23 (45.1%) were male. Since evaluating urinary incontinence and voiding-related complaints in pediatric patients is physiologically appropriate after the age of five, the age range for the study population was determined to be 6–17 years. The mean age of the patients was 11.8 (± 3.8) years. The average disease follow up duration in the study population was determined to be 45.4 months (ranging from 2 to 79 months). The evaluation of bladder function in patients was conducted during routine follow-ups while they were in remission. Patients without urological clinical symptoms underwent a single urological assessment, and since they exhibited no symptoms, no repeat follow-up examinations were performed. Throughout the study period, none of the patients presented with repeat any urological complaints. No abnormal findings were detected in the complete urine analysis of all patients included in the study. In the evaluation of urinary system ultrasonography, hydronephrosis was detected in 3 (5.9%) patients. Two of the patients were male, and one was female. The first patient (14 years, M) had bilateral mild hydronephrosis (Grade 1), the second patient (17 years, M) had mild hydronephrosis (Grade 1) in the right kidney. Additionally, in the second patient, there was a minimal increase in renal parenchymal echogenicity suggestive of renal parenchymal disease. The third patient (12 years, F) had grade 1 hydronephrosis in the right kidney with measured renal pelvis anteroposterior diameter of 8.6 mm. The average bladder wall thickness (BWT) measured on ultrasonography for all patients was 1.14 (± 1.6) mm. The comparison of bladder wall thickness according to the patients’ gender is presented in Table 1. There was no statistically significant difference in bladder wall thickness values based on the patients’ gender (*p* > 0.05).

**Table 1:** Comparison of gender according to Ultrasonography – Bladder wall thickness values

	Gender	*N*	Mean	Std. Deviation	*p*
USG-BWT	Female	28	1.31	2.19	0.414
	Male	23	0.92	0.671	

*independent t test

A total of 31 patients with measurable post-micturition residual urine (PMR) during ultrasonographic evaluation were identified. Among these patients, 15 were male and 16 were female. Significant PMR measurements (> 20 ml) were recorded in 15 (29.4%) patients, with 7 males and 8 females.

The comparison of measured bladder capacities (BC) with expected bladder capacities (EBC) based on uroflowmetric and ultrasonographic results is presented in Table 2. While there was no statistically significant relationship between EBC and USG-BC values, a statistically significant positive correlation was found between EBC and UFM- BC values (r: 0.466; *p* < 0.001). When examining Qmax values, normal Qmax values were observed in 9 (17.6%) patients (6 F/3 M).

**Table 2:** Comparison of Uroflowmetry - Bladder capacity and ultrasonography -Bladder capacity values according to EBC (*n* = 51)

		EBC
USG-BC	r	0.061
	p	0.672
UFM-BC	r	0.466^**^
	p	< 0.001

*spearman correlation analysis

## Discussion

Studies on familial Mediterranean fever generally focus on its epidemiology, pathophysiology and molecular mechanisms, clinical findings, and treatment outcomes. These studies often aim to investigate the urinary system complications of FMF and the frequency of bladder involvement. Although bladder involvement in these patients is rare, it can occur, typically associated with inflammation, and may lead to more severe symptoms when accompanied by additional factors such as urinary tract infections [10–11].

Bladder symptoms in this patient group may manifest as urinary tract infections, bladder irritability, or dysfunction. Pyuria, bacteriuria, hematuria, proteinuria, and crystalluria are urinary analysis findings observed, particularly during attacks, in children diagnosed with FMF [12]. In our patients, all had normal findings on complete urine analysis, likely attributed to regular colchicine use and the low frequency of attacks.

Cases of renal failure due to amyloidosis accumulation have been reported in children diagnosed with familial Mediterranean fever [13]. Our patient group consists of patients without renal and systemic amyloidosis. For these reasons, no kidney anomalies were reported in the ultrasound screening of our patients. While in our series, hydronephrosis was detected in 3 (5.9%) patients, additionally, a minimal increase in parenchymal echogenicity was observed in one (1.9%) patient. When obstruction in the urinary system is mentioned, it is quite natural to think of hydronephrosis and accompanying obstruction. However, there are pathologies that can cause hydronephrosis without obstruction. Pathologies such as vesicoureteral reflux cause dilation of the urinary system without any obstruction in any part. Therefore, not every narrowing leading to hydronephrosis may constitute an obstruction. If renal damage does not occur, it can be interpreted that the kidney successfully defended itself and adapted. However, waiting for renal damage to occur to understand whether a narrowing causing hydronephrosis is a real obstruction is unacceptable. Therefore, close monitoring of at-risk kidneys in patients diagnosed with FMF is crucial. In this context, when considering our results, close ultrasonographic monitoring of the urinary system seems important.

FMF is known to be an inflammatory disease. Therefore, systemic inflammation can also cause changes in bladder tissue. Bladder involvement in amyloidosis is rare, and involvement is mostly limited to the bladder, termed primary bladder amyloidosis [6]. Secondary bladder amyloidosis has been reported in diseases such as rheumatoid arthritis, Crohn’s disease, ankylosing spondylitis, multiple myeloma, and familial Mediterranean fever [7]. The study population consisted of patients who were under routine follow-up, in remission, and receiving colchicine treatment. Our patients were closely followed up (at three-month intervals) by our pediatric rheumatologist (Professor Betül Sözeri). It was confirmed by her data that none of the patients in this study had systemic or urinary system amyloidosis.

Based on the findings of this study, it is hypothesized that the observed effects on the bladder may be related to a possible systemic inflammatory response. As with all systemic diseases, it is well known that systemic inflammatory responses continue to exert effects on various organs even when the patient is in remission. In pediatric patients undergoing growth and development, this chronic inflammatory process may have an impact on the bladder, which possesses a highly complex muscular structure and neuronal transmission network.

In our series, bladder wall thickness measured on urinary system ultrasonography was evaluated. However, there is no specific data in the literature on this because there are limited studies on the effects of FMF on the bladder. The bladder wall thickness in a voided bladder should be less than 3 mm in healthy individuals [14]. In our study, the average BWT in the patient group was measured as 1.14 (± 1.6) mm and was found within normal limits. Additionally, no effect based on gender was detected. However, if there are concerns regarding bladder wall thickness when a child is diagnosed with FMF or during treatment, evaluation by a pediatric urologist or pediatric nephrologist and appropriate imaging tests are recommended. This way, any potential bladder pathology or inflammatory changes can be identified early, and appropriate treatment can be planned.

In children diagnosed with FMF, post-micturition residual urine values may generally be within the normal range, but this is not always the case. Additionally, ultrasonographic measurement of bladder capacity may be misleading in the evaluation of bladder capacity. Therefore, it is important to evaluate PMR values and monitor them closely in termsofbladderfunction. Some authors have defined pathologically a PMR of more than 20 ml or a residual urine volume of more than 10% of estimated bladder capacity after repeated voiding without bladder overdistension [15]. Ultrasonographic and uroflowmetric measurements are two effective methods for evaluating bladder capacity in children. Bladder volume measurement by ultrasonography is based on length, width, and height measurements of the bladder’s widest dimensions. The obtained volume value is used to determine bladder capacity. This value reflects the maximum amount of urine a normal bladder can typically hold [16]. Automated volume measurement modes, characterized by the highest measurement accuracy, are applied when measuring bladder volumes and assessing the residual urine volume after urination. (Toschiba Aplio 500) In most cases, these methods are based on measurements made using the prolate ellipsoid formula. Measurements must be taken in two planes perpendicular to each other at the maximum sections of the bladder [17]. It should be noted that the results are subject to unavoidable calculation errors [18–19]. In our study, urinary ultrasonography was performed by a single radiologist, and measurements were taken following the same principles. We believe this approach enhanced the reliability of our results and increased the impact on the accuracy of the findings. Altough considering the high reliability of our results in this context, we have observed that evaluations using ultrasonography can sometimes be misleading. Additionally, in routine daily follow-ups, the involvement of different radiologists, the use of different ultrasonography devices, variations in bladder filling levels, and differences in measurement locations could further reduce the reliability of the results. Therefore, we recommend that patients be evaluated with these factors in mind.

Uroflowmetric measurement, on the other hand, is performed using a special device called a uroflowmeter. Uroflowmetry is a test used to measure a child’s urine flow rate, duration, and volume. In our clinic, the uroflowmeter device used (MMS-Medical Measurement Systems Solar Uro 1010288730) does not allow PMR measurement. Both methods complement each other and are selected and interpreted by a pediatric urologist based on the individual situation of the child.

In our study, a significant positive correlation was found between bladder capacity measured with uroflowmetry and expected bladder capacity. Patients’ bladder capacities measured with uroflowmetry were higher. This finding suggests possible changes in bladder structure due to inflammation.

Qmax values in children are determined based on data obtained during uroflowmetry testing. Qmax is the most appropriate quantitative parameter used to evaluate bladder outlet obstruction. Q max value represents the maximum urinary flow rate during micturition. The measurement of Q max is a highly informative parameter for conditions such as the patency of the urethral voiding pathway and bladder contraction function. “Normal” maximum urine flow rate (Qmax) in children can vary depending on age, gender, and other factors. The measured flow rates in children were first published by Kaufman [20]. Normal flow rates were estimated to be between 13 and 26 ml/s, with the lowest voiding volume estimated to be 1.5 ml for children. Scott and McIlhaney [21] measured maximum flow rates of 8–29. However, generally, typical Qmax values in children are lower than in adults. Flow rates between 5 and 15 ml/s are generally considered normal in children. It has been reported that there is a linear correlation between Qmax and the square root of voided volume in both normal children and adults. If the square of Qmax is equal to or greater than the voided volume, the recorded Qmax is likely normal. Urinary flow rate is now generally considered the most useful parameter in determining an individual’s voiding ability [22–23]. In our series, normal Qmax values were observed in 17.6% of patients. This ratio indicates that voiding ability was impaired in 82.4% of patients. Despite the absence of clinical complaints, it seems important for the follow-up physician to be informed about these measurement values and to consider close monitoring of these patients. If necessary, further investigation should be carried out.

Urodynamic evaluation is the reference diagnostic method for determining lower urinary tract dysfunction. However, due to its invasive nature, especially in the pediatric age group, its application is not easy. Ultrasonographic measurement of bladder wall thickness can be used as a complementary tool in the assessment of functional lower urinary tract disorders, treatment selection, evaluation of treatment response, and follow-up process.

Lower urinary tract dysfunctions in children can be a dynamic group of diseases that change over time and can present with different symptoms and signs. Urodynamic study findings may vary between disease groups, and individual differences may be observed even within the same disease group. Moreover, changes can occur in the same patient during follow-up. Similarly, children are organisms undergoing growth and maturation. Therefore, it would be appropriate to evaluate each case individually rather than confining patients and findings to limited patterns.

Given that FMF is a systemic disease characterized by chronic inflammation, we think that inflammatory effects affect the all systems and organs also bladder. Considering the bladder’s muscular structure and neuronal innervation pathways, it is a complex and dynamic organ. Therefore, it is quite likely that it is affected by inflammatory processes.

There are some limitations to our study; lack of electromyelography (EMG) in uroflowmetry, the inability to measure PMR in uroflowmetry, and the inability to compare with voiding diary evaluations.

In addition to these, assessing the difference between the time of diagnosis and the current follow-up period would indeed be interesting and noteworthy. However, since this study includes only a single urological evaluation and patients did not undergo a urological examination at the time of diagnosis, this represents a limitation of our study.

We initially considered asking patients to complete a micturition diary, which would track volume and frequency. However, given that our patients did not have urological clinical complaints, we felt that this method might be somewhat disruptive to their daily routine (requiring measurements over at least two days). Therefore, we prioritized the evaluation of bladder volume through uroflowmetry and bladder ultrasonography. Of course, having micturition diary data would have strengthened our study. We consider the absence of this evaluation as one of the limitations of our study.

The absence of a healthy control group is one of the significant limitations of our study. When evaluating the stability or worsening of bladder function in patients during long-term follow-up, the lack of comparative data prevents us from making definitive conclusions about the disease progression. However, our long-term follow-up data will guide us in strengthening the results of our study.

With these data, it is important that patients are more carefully questioned regarding urological symptoms during their routine follow-ups in the pediatric rheumatology department. If necessary, based on clinical monitoring, they should be referred for further evaluation to the pediatric urology outpatient clinic. It is also recommended that patients be thoroughly informed during the long-term treatment of such systemic diseases and that detailed systemic examinations be conducted, with a comprehensive anamnesis that includes all organ systems. This approach will help with the early diagnosis of potential organ dysfunctions in later stages, enabling a preventive approach.

In conclusion, the evaluation of bladder function in children with FMF who are asymptomatic for urological symptoms using parameters measured in uroflowmetry is important. PMR values may generally be within the normal range, but this is not always the case. Additionally, ultrasonographic measurement of bladder capacity may be misleading in the evaluation of bladder capacity.

## Data Availability

The datasets analyzed in this study are available from the corresponding author upon reasonable request.
